# Micro- and nanochamber array system for single enzyme assays

**DOI:** 10.1038/s41598-023-40544-4

**Published:** 2023-08-16

**Authors:** Kazuki Iijima, Noritada Kaji, Manabu Tokeshi, Yoshinobu Baba

**Affiliations:** 1 Department of Biomolecular Engineering, Graduate School of Engineering, Nagoya University, Furo-cho, Chikusa-ku, Nagoya, 464-8603 Japan; 2 Department of Applied Chemistry, Graduate School of Engineering, Kyushu University, 744 Motooka, Nishi-ku, Fukuoka, 819-0395 Japan; 3 Institute of Nano-Life-Systems, Institutes of Innovation for Future Society, Nagoya University, Furo-cho, Chikusa-ku, Nagoya, 464-8603 Japan; 4 Division of Applied Chemistry, Faculty of Engineering, Hokkaido University, Kita-13, Nishi-8, Kita-Ku, Sapporo, 060-8628 Japan; 5 Institute for Quantum Life Science, Quantum Life and Medical Science Directorate, National Institutes for Quantum Science and Technology, Chiba, 263-8555 Japan; 6 School of Pharmacy, College of Pharmacy, Kaohsiung Medical University, 100, Shih-Chuan 1st Rd., Kaohsiung, 807 Taiwan, ROC

**Keywords:** Lab-on-a-chip, Biochemical assays

## Abstract

Arrays of small reaction containers, ranging from 624 femtoliters (10^–15^ L) to 270 attoliters (10^–18^ L), for capturing a single enzyme molecule and measuring the activity were developed along with a new reversible sealing system based on a pneumatic valve actuator made of polydimethylsiloxane (PDMS). The valve was actuated by PBS solution, effectively preventing evaporation of the solution from the micro- and nanochambers and allowing the assay to be performed over a long period of time. The hydrolysis rates of *β*-D-galactosidase (*β*-gal), *k*_*cat*_, were decreased according to the decrease of the chamber size, and the overall tendency seems to be symmetrically related to the specific surface area of the chambers even under the prevented condition of non-specific adsorption. The spatial localization of the protons in the chambers, which might could affect the dissociation state of the proteins, was also investigated to explain the decrease in the hydrolysis rate. The developed chamber system developed here may be useful for artificially reproducing the confined intracellular environment and molecular crowding conditions.

## Introduction

Methodological developments in microscopy and related techniques have provided new insights into enzymology at the level of a single enzyme molecule to understand what causes static heterogeneity and the dynamic fluctuation arise from and how they affect the activity. For this implementation, different methodological approaches have been proposed to isolate a single enzyme molecule with enough substrates to observe the activity. A simple approach has been carried out using highly diluted enzyme solution in capillary electrophoresis (CE)^1,2^, but CE only confines a reaction space two-dimensionally and is a continuum in the direction of flow, which limits time-resolved observation. Therefore, a three-dimensional confinement such as liposomes is essential for accurate measurements in enzymology^3^. If we focus on an outer boundary of a small reaction space containing a single enzyme molecule, it can be classified into three systems: liquid–liquid, liquid–solid, and liquid–air systems. The liquid–air system is well known as an airborne particle but is not suitable for this purpose due to its fast and random movement and fast and easy evaporation. Therefore, liquid–liquid and liquid–solid systems have been mainly proposed as a reaction container and used for single molecule enzymology.

The first approach to a liquid–liquid system was demonstrated using droplets of a water-in-oil emulsion obtained with a nebulizer consisting of a capillary tube of approximately 20 µm diameter^4^. The activities of individual *β*-D-galactosidase (*β*-gal) encapsulated in 14–15 µm diameter droplets with a fluorogenic substrate were measured. The emulsion-defined femtoliter droplet format, equivalent to 10 cubic microns, gave rise to the idea of the next-generation genome sequencing and made a major contribution to biology^5^. However, standard emulsification methods produce droplets with a broad size distribution and spontaneous fusion/fission occurs. To suppress the broad size distribution and improve the long-term stability, an amphiphilic molecule (a surfactant) is added. Accurate measurements of a single enzyme activity become increasingly difficult because the surfactant can cause conformational changes on the enzyme as well as activity changes of water. Another approach to liquid–liquid systems is liposomes, which consist of unilamellar vesicles ranging in size from 100 nm^6,7^ to a few µm^8,9^ in diameter and provide the more biologically relevant environment. An ultimate ultra-small biologically relevant confinement is the viral capsid, which has an internal diameter of a few nm, equivalent to a few zL, and a single horseradish peroxidase enzyme is encapsulated inside the capsid and its activity is studied^10^. These simple approaches to mimic the interior of cells or viruses are very useful for understanding not only in vivo enzyme kinetics but also the condensation of biomolecules in living cells. Biomolecular condensates in cells have demonstrated a range of diverse cellular functions through associative and segregative phase transitions, e.g. transcription regulation^11^ and protein circuit modulation^12^, the methodology at micro- to nanoscale enzymology could offer a variety of options to focus on their research objectives through easy size and time control^13^.

On the other hand, Yeung et al. challenged to fabricate small chamber arrays consisting of about 135 fL, 5–12 µm in diameter and 4–6 µm in height, on thin fused-silica glass, and then, tightly attached to flat quartz plates for encapsulation of single enzyme molecules^14^. This approach, which can be categorized as a liquid–solid system, has been widely applied to single-molecule enzymology through improvements in plate materials and encapsulation methods. Arrays of small reaction containers, ranging from femtoliter (10^–15^ L) to attoliter (10^–18^ L), for single-molecule enzymology have been fabricated using optical fiber bundles^15,16^, polydimethylsiloxane (PDMS)^17,18^, and fused silica slides^19,20^. In this liquid–solid system, individual enzyme molecules are encapsulated in the small reaction containers using a dilute solution, so that the number of reaction containers is much greater than the number of enzyme molecules present. The most critical issue in this system is how to seal the liquid to the solid surface without leaking and evaporating the liquid during the experiments. Although it is difficult to achieve perfect containment with homogeneous materials such as quartz on a quartz plate, flexible and self-adhesive polymers such as PDMS are suitable materials. Femtoliter chamber arrays have been developed on top of optical fiber bundles sealed by mechanical force with silicone gaskets^15,16,21,22^, and later an oil sealing method has been applied to ensure a tight seal and avoid evaporation of the reaction solution^23^. Similar oil sealing approaches have also been carried out for PDMS chambers^24^, but the various problems associated with the liquid interface mentioned above cannot be avoided.

Here, we used a pneumatic valve to confine individual enzyme molecules in small reaction containers ranging in volume from femtoliter to attoliter to study the fundamental enzymology in confinement. Although similar approaches have been demonstrated with the dimple machine^20^ and the air-operated valve^25^, we operated the pneumatic valve with PBS solution to prevent the solvent evaporation even during prolonged observation and demonstrated a single *β*-gal assay in different chamber sizes ranging from 10 µm to 800 nm in diameter, which was expected to provide more experimentally accurate and reproducible values of *β*-gal activity at the single molecule level were expected to obtain compared to our previous work^26^. The reusability of the device could contribute to accurate and reproducible measurements that require delicate handling. The main reason for choosing *β*-gal as a target enzyme was its appropriate turnover number to observe (not too fast, not too slow) and its robustness to the surrounding environment especially oxygen compared to reductases such as diaphorase^27,28^. The proton concentration in these small chambers, which indicates the location of the proton, could affect the hydrolysis reaction and was therefore investigated using a pH-sensitive fluorescent dye, Carboxy SNARF-1. The developed solid-surface enclosed system automatically acquires a long-term observation data automatically from femtoliter to attoliter scale chambers and can be simplify the discussion on the interfacial molecular interaction.

## Materials and methods

### Enzyme kinetic assays in the bulk

FDG, *β*-D-galactosidase (*β*-gal), fluorescein, 2-mercaptoethanol were purchased from FUJIFILM Wako Pure Chemical Corporation (Osaka, Japan). A phosphate buffer (PB, pH = 7.4) was purchased from Sigma-Aldrich, Japan (Tokyo, Japan). The basic assay solution was prepared by mixing *β*-gal and FDG (50, 100, and 200 µM) in a 100-mM phosphate buffer (pH 7.5) containing 1 mM MgCl_2_ and 0.2% (v/v) 2-mercaptoethanol. Fluorescence intensity was monitored using a fluorescence spectrophotometer (FP-6500, JASCO Corporation, Tokyo, Japan) at an excitation/emission wavelength of 490 nm/520 nm and a temperature of 30 °C.

### Device fabrication

**Figure 1:**
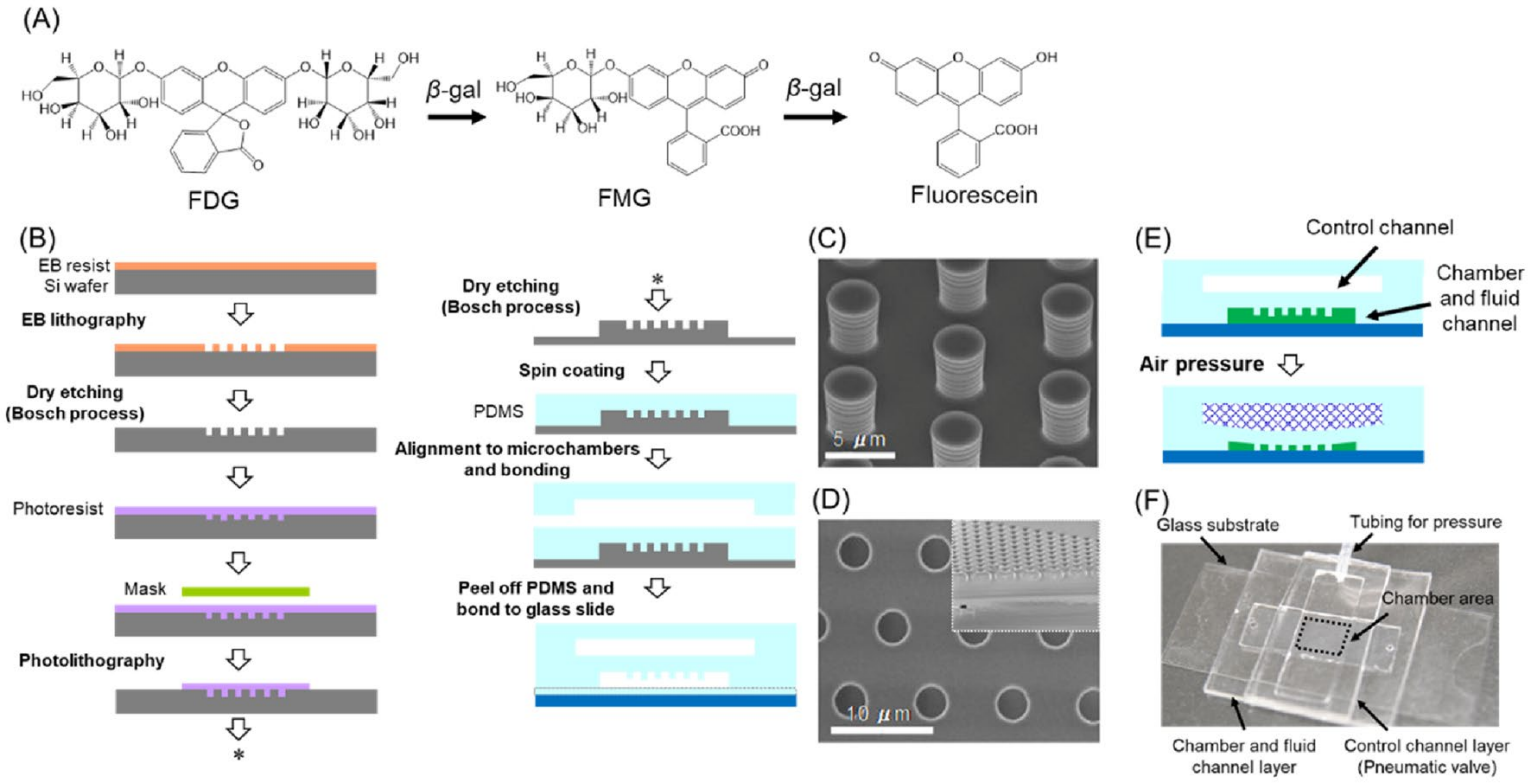
(**A**) Hydrolysis reaction scheme by β-gal used in this study. (**B**) Fabrication process of the micro and nanochambers equipped with a pneumatic valve. (**C**) A scanning microscope (SEM) image of dry-etched Si mold for the microchambers. (**D**) A SEM image of PDMS microchambers and a cross section image (insert). (**E**) Working principle of the microchambers with pneumatic valves. (**F**) Overview of the device.

The polydimethylsiloxane (PDMS)-based microfluidic devices were fabricated using standard soft lithography methods^29^. The master moulds of the micro- and nanochamber patterns were fabricated on the silicon substrate by electron-beam (EB) lithography and Bosch etching; the fabrication process is shown in Fig. 1. A thick (~ 200 nm) positive-EB resist (ZEP-520, Zeon Corp.) was spin-coated at 600 rpm for 120 s followed by 2000 rpm for 5 s, and then, post baked at 180 °C for 3 min, after which the pillar pattern was delineated by EB lithography. After EB resist development, the pillar patterns were dry etched using the Bosch deep reactive ion etching (DRIE) process. Acetone was used to remove the EB resist. A thick (~ 2 µm) photoresist (OFPR-800, Tokyo Ohka Kogyo Co., Ltd.) was spin-coated on the pillar-patterned substrate at 500 rpm for 5 s followed by 1500 rpm for 120 s, and then, post baked at 95 °C for 3 min, and then the wide microchannel patterns on the outside of the pillar patterns were exposed through the Cr mask. After the photoresist was developed, the fluid channel patterns were dry-etched by the DRIE process. The fabricated master mould was silanised in a desiccator containing trichloro(1H,1H,2H,2H-perfluorooctyl)silane (Sigma-Aldrich Co. LLC., Tokyo, Japan) vapor before adding a mixture of the curing agent and PDMS prepolymer (SYLGARD 184 Silicone Elastomer Kit, Dow Corning Toray Co., Ltd., Tokyo, Japan) in a ratio of 1:10. The prepolymer mixture was degassed for 2 h in a vacuum desiccator and then cured for 2 h at 80 °C. The PDMS replica was peeled from the master and the surface of the PDMS replica was treated with a soft plasma etching device (SEDE-PFA, Meiwafosis Co., Ltd., Tokyo, Japan) for 90 s at 5 mA immediately before use. As a pneumatic valve, the control channels for air pressurisation and sealing of the micro- and nanochambers were fabricated using the same fabrication process as the fluid channel. In this study, twelve different sizes of micro- and nano chambers were fabricated, ranging from 270 aL(ϕ:800 nm × H:600 nm) to 624 fL(ϕ:10 µm × H:10 µm), were fabricated as shown in Figure S1.

### Single-enzyme kinetic assays in microchambers

The surface of the cover slip was spin-coated with uncured PDMS for 60 s at 3000 rpm. The PDMS was then cured for several hours at room temperature (20–25 °C). A mixed solution of *β*-gal and FDG was introduced into the fluid channel from the reservoir. The pneumatic valve enclosed the assay solution in the micro- and nanochambers by applying air pressure to the control channel, as shown in the supporting movie. The spontaneous adhesion of the PDMS ensured the perfect sealing of the solution in the micro- and nanochamber. The enclosed micro and nanochamber device was placed on the stage of the total internal reflection fluorescence microscope (TIRFM, IX71, Olympus, Tokyo, Japan) that was equipped with an argon-ion laser (488 nm), after which the fluorescence in the microchambers was observed through high magnification objectives (UAPON150 × OTIRF, UPLAPO100 × OHR, UPLAPO60 × OHR) under evanescent wave illumination. All the observations were performed at 30 °C using a stage-top incubator (TOKAI HIT Co., Ltd., Fujinomiya, Japan). Fluorescence images were captured by an EB-CCD camera (C7190, Hamamatsu Photonics)^30^ coupled to an image intensifier unit (C8600, Hamamatsu Photonics, Hamamatsu, Japan) and analysed using image analysis software (Aquacosmos, Hamamatsu Photonics).

To suppress non-specific adsorption on the PDMS surface, 2-methacryloyloxyethyl phosphorylcholine polymer (MPC polymer, a gift from Prof. Kazuhiko Ishihara, Department of Bioengineering, The University of Tokyo) and poly(N-hydroxyethyl acrylamide) (PHEA, a gift from Prof. Annelise E. Barron, School of Engineering and Medicine, Stanford University) were used for the PDMS-based surface coating.

### Evaluation of a pH gradient in the micro and nanochambers

A pH indicator, 5-(and-6)-carboxy SNARF-1 dye, was purchased from Thermo Fisher Scientific K.K. (Tokyo, Japan). To measure the pH of the solution encapsulated in the micro- and nanochambers, the ratio of the fluorescence intensities of the dye at two emission wavelengths, 580 nm and 640 nm, excited at 488 nm, was used by a confocal microscope (FV1000, Olympus, Tokyo, Japan) was used for quantitative determinations of the pH.

## Results and discussion

### Micro and nanochambers equipped with a pneumatic valve

As shown in Fig. 1B–F, the chamber and the fluid channel layer were first placed on the glass slide with a thin layer of spin-coated PDMS, and then, the control channel layer was stacked. To investigate the working principle of the pneumatic valve for sealing the micro- and nanochambers, the syringe filled with phosphate buffer was connected to the inlet hole of the control channel via a silicone tube and the phosphate buffer was introduced into the control channel. This control channel has an inlet hole but no outlet hole and was driven by buffer solution to maintain the appropriate pressure and the buffer-soaked PDMS chambers for long-term observation. The gas permeability of PDMS itself has been extensively studied as well as hydrophobic liquid for pervaporation separation^31–33^, but the permeability of water or water-soluble substrates used in this study has not been demonstrated. Migration of water or solutes may be possible but the rate should be much slower compared to the measurement time in this experiment and the influence could be ignored. Supporting movie S1 shows how 0.1 µM fluorescein solution was enclosed in the 264-fL chambers (*ϕ*:9.1 µm × *H*:6.3 µm) and no fluorescein leakage was observed. Therefore, the following enzymology experiments were performed using the valve-equipped micro- and nanochambers.

**Figure 2:**
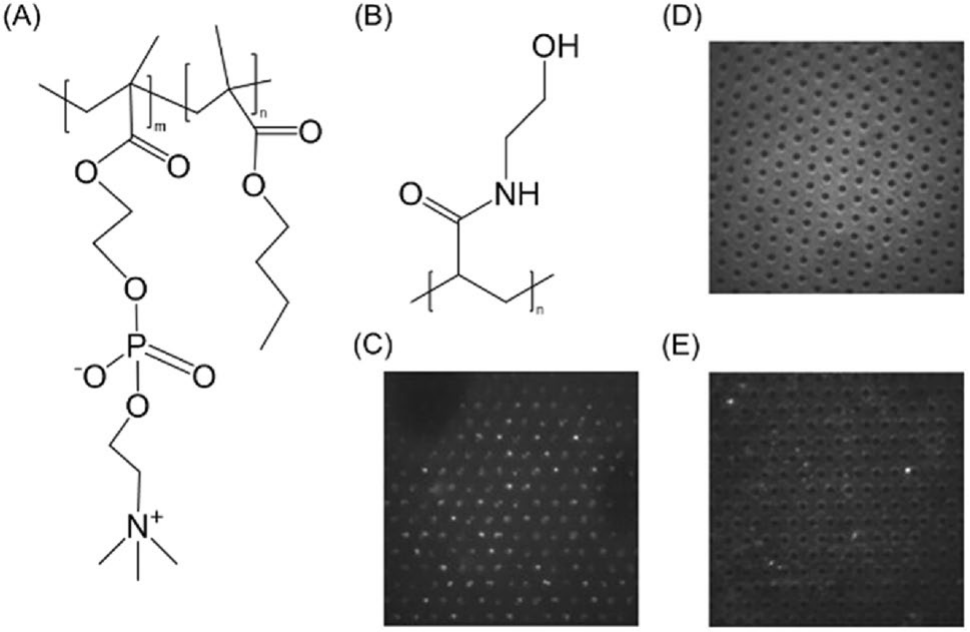
Chemical structures of (**A**) Poly(2-Methacryloyloxyethyl phosphorylcholine-*random*-n-butyl methacrylate) and (**B**) Poly(N-hydroxyethyl acrylamide), which are used for the dynamic coating of the microchambers. Fluorescent microscope images of the PDMS chambers with (**C**) non-coating, (**D**) MPC polymer, and (**E**) PHEA after 10-min incubation of 1 µg/mL FITC-BSA and washing by phosphate buffer.

As we described in the previous study^26^, adsorption of enzymes encapsulated in the chambers to the chamber surface is critical in measurements for hydrolysis rate measurements. The hydrophilicity of the original PDMS surface is relatively high and causes denaturation of protein molecules including enzymes through hydrophobic interactions^34^. Poly(2-methacryloyloxyethyl phosphorylcholine-*random*-n-butyl methacrylate) (MPC polymer) and poly(N-hydroxyethylacrylamide) (PHEA), shown in Fig. 2, were used as dynamic coating reagents to prevent non-specific adsorption of the enzymes and the substrates. MPC polymer was added to the chamber and fluid channel and incubated for 24 h at room temperature. PHEA was added to the chamber and fluid channel and incubated for 1 h at room temperature after washing with MilliQ water and 1 M HCl solution for 15 min each. Both of polymers were coated after assembly of the device. To confirm the surface coating, the chambers were filled with 1 µg/mL of FITC-BSA for 10 min, washed with phosphate buffer and then observed by fluorescence microscopy. In this study, considering the future application of the single enzyme assay to other than *β*-gal, FITC-BSA was used to trace the adsorption behavior because BSA is widely used in biochemistry and molecular biology experiments. As shown in Fig. 2D,E, both of coatings sufficiently suppressed the non-specific adsorption of FITC-BSA but the MPC polymer increased the background fluorescence level despite the location. Therefore, MPC polymer was not used in the following enzymology experiments because we do not have a clear answer to explain the phenomenon.

### Enzyme kinetic assays in bulk

The time course of the fluorescence intensity of fluorescein produced by *β*-gal was measured by UV–VIS spectrophotometer under different concentrations of substrate, FDG, concentrations as ensemble-averaged enzyme kinetics. The hydrolysis reaction of *β*-gal consists of two steps, FDG to FMG and FMG to fluorescein, and the first hydrolysis rate is known to be significantly slower than the second hydrolysis rate. Therefore, the first hydrolysis rate is a rate-limiting step and corresponds to the observed hydrolysis rate. In this study, this apparent hydrolysis rate was assumed to be the hydrolysis rate, *k*_*cat*_. The Michaelis–Menten constant *K*_M_ and the hydrolysis rate *k*_*cat*_ were calculated according to previous methods^26,35^. The measurements, as shown in Figure S2, yielded *K*_M_ = 56.5 µM and *k*_*cat*_ = 55.1 s^–1^, which is three times higher than our previous reports of 18.0 s^–1^. Quantitative analysis of an enzyme activity is a sensitive experiment, and often performed by the same enzyme from different batches is assayed at different times, so a large inter-laboratory variation of the absolute value was often observed. Even in a single run, a large coefficient of variation exceeding 100% has occasionally been observed^36^. Experimental imperfections, such as slight differences in temperature and buffer concentration, could be responsible for the variable data. However, our focus was on the effect of reducing the size of the reaction space on the enzyme activity and not on the absolute value of the activity, and the following chamber experiments were performed with great care to suppress the variation.

### Single enzyme kinetic assays in micro- and nanochambers with a pneumatic valve

Twelve different sizes of micro- and nanochambers, ranging from 270 aL(ϕ:800 nm × H:600 nm) to 624 fL(ϕ:10 µm × H:10 µm), were used for a single *β*-gal kinetic assay. The concentration of FDG and *β*-gal in the phosphate buffer and the incubation time were optimized according to the chamber sizes to capture the single *β*-gal molecule with enough FDG to maximize the hydrolysis rate. The typical three results of the single enzyme assay using 624-fL, 61-fL, and 270-aL chambers are shown in the main figures and the remainder are shown in the supplemental figures. To avoid photobleaching of the product, fluorescein, an excitation light shutter was kept closed except for the fluorescence image acquisition time. Crosstalk of the fluorescence light could be minimized by optimizing the incubation time and the excitation laser power even in the smallest 270-aL nanochamber array as shown in Fig. 5.

**Figure 3:**
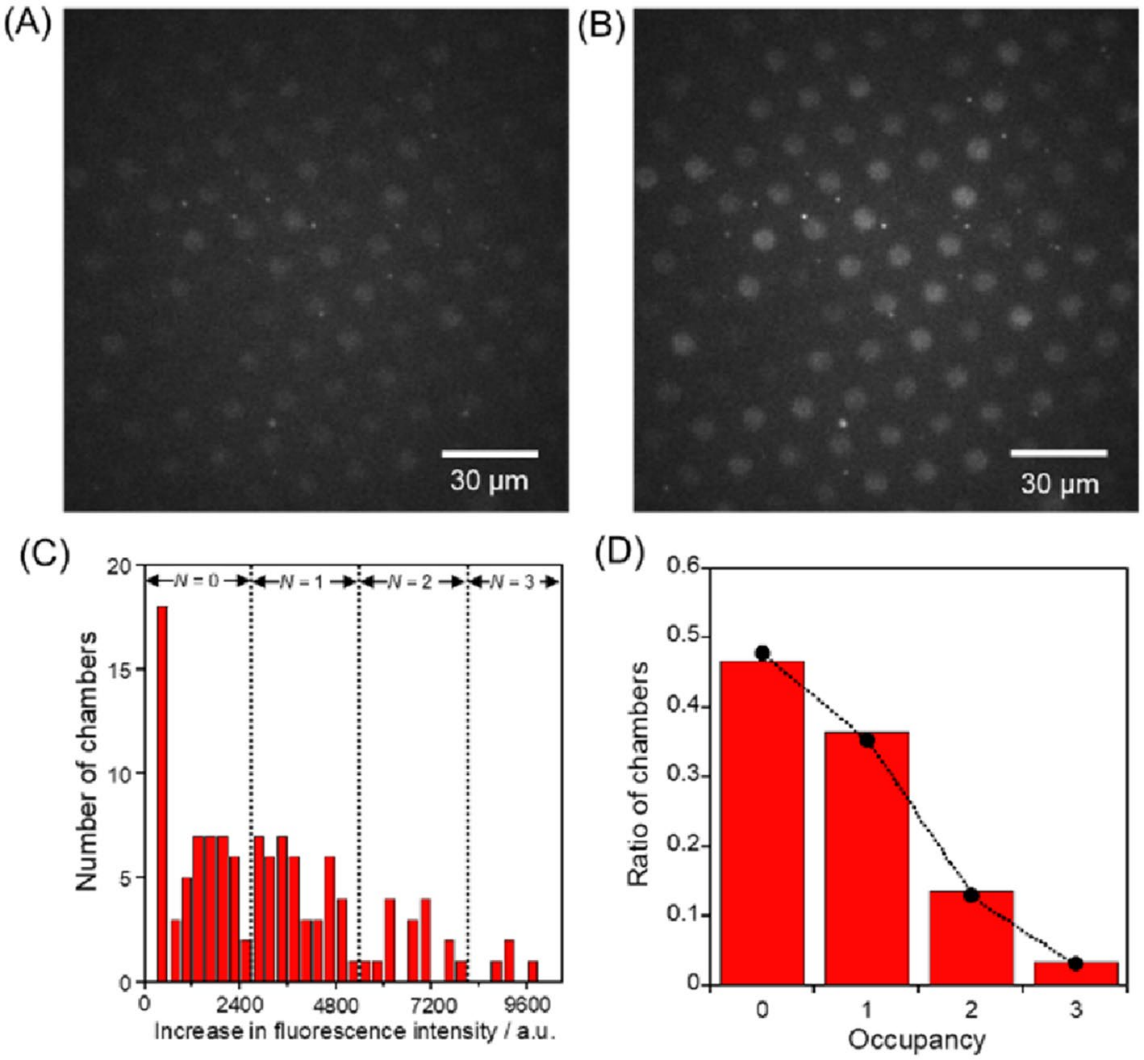
Single-enzyme assay in the 624-fL chambers. The fluorescent images of the microchamber array enclosing *β*-gal after times (**A**) 0 and (**B**) 2 min. (**C**) Histogram of the fluorescent intensity changes for 2 min. The concentrations of *β*-Gal and FDG were 2.0 ng/mL and 200 μM, respectively. The dotted lines indicate the thresholds of fluorescence intensity increase with different numbers of *β*-gal molecules in the microchambers, assuming a Poisson distributed encapsulation of *β*-gal molecules in the microchamber. (**D**) Occupancy distribution of the microchambers under the condition of 2.0 ng/mL *β*-gal. The bars show the ratio of the microchambers with an occupancy of *N* enzymes (*N* = 0, 1, 2, 3). The circles indicate the probability of the ratio of the microchambers that were captured *N* enzymes at λ = 0.737, assuming it was a Poisson distribution. The detail explanation is described in the main text. All the fluorescent images were captured under the illumination of a 72.7 μW excitation laser.

**Figure 4:**
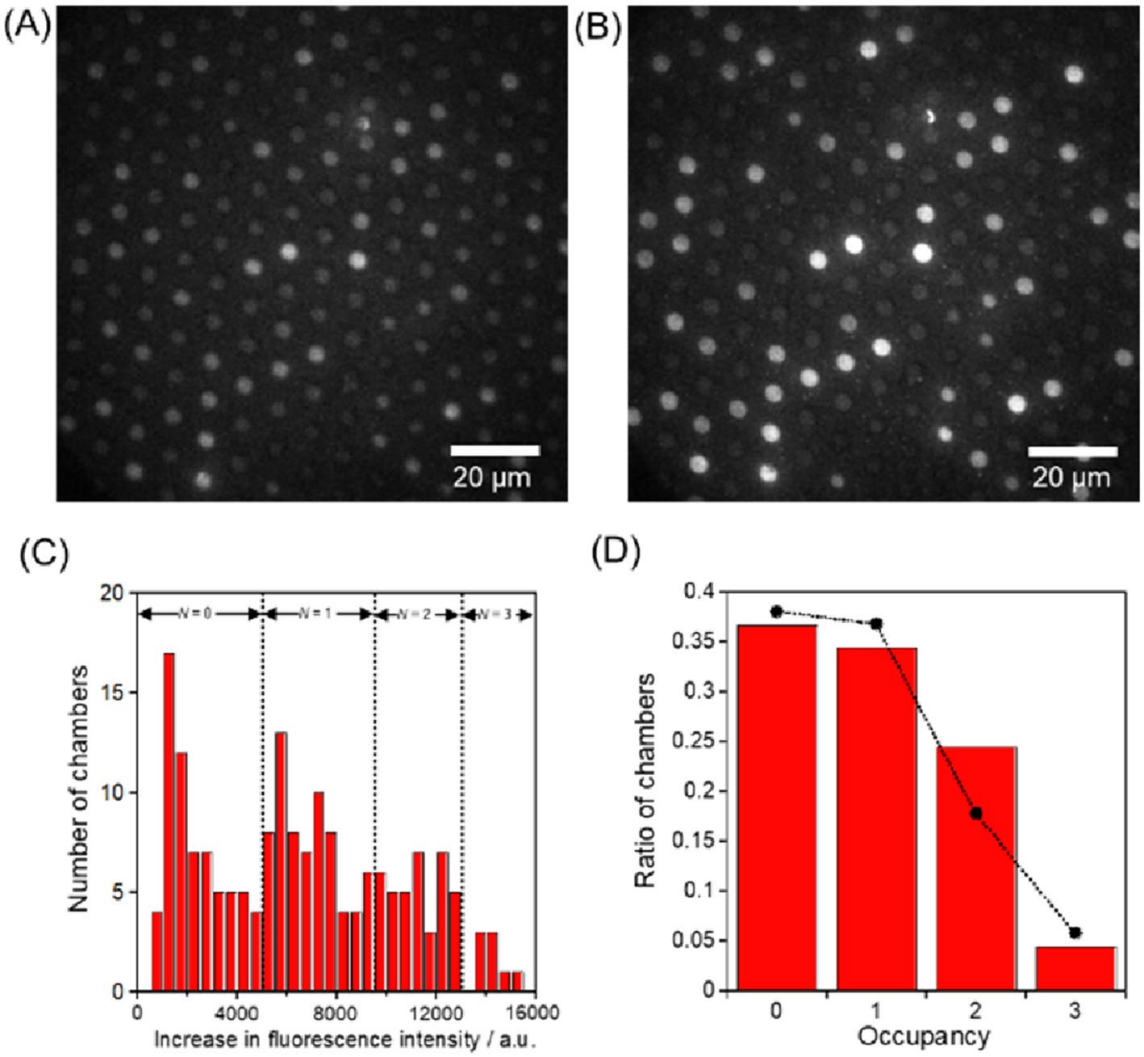
Single-enzyme assay in the 61-fL chambers. The fluorescent images of the microchamber array enclosing *β*-gal after times (**A**) 0 and (**B**) 2 min. (**C**) Histogram of the fluorescent intensity changes for 2 min. The concentrations of *β*-Gal and FDG were 6.0 ng/mL and 200 μM, respectively. The dotted lines indicate the thresholds of fluorescence intensity increase with different numbers of *β*-gal molecules in the microchambers, assuming a Poisson distributed encapsulation of *β*-gal molecules in the microchamber. (**D**) Occupancy distribution of the microchambers under the condition of 6.0 ng/mL *β*-gal. The bars show the ratio of the microchambers with an occupancy of *N* enzymes (*N* = 0, 1, 2, 3). The circles indicate the probability of the ratio of the microchambers that were captured *N* enzymes at λ = 0.966, assuming it was a Poisson distribution. The detail explanation is described in the main text. All the fluorescent images were captured under the illumination of a 17.8 μW excitation laser.

**Figure 5:**
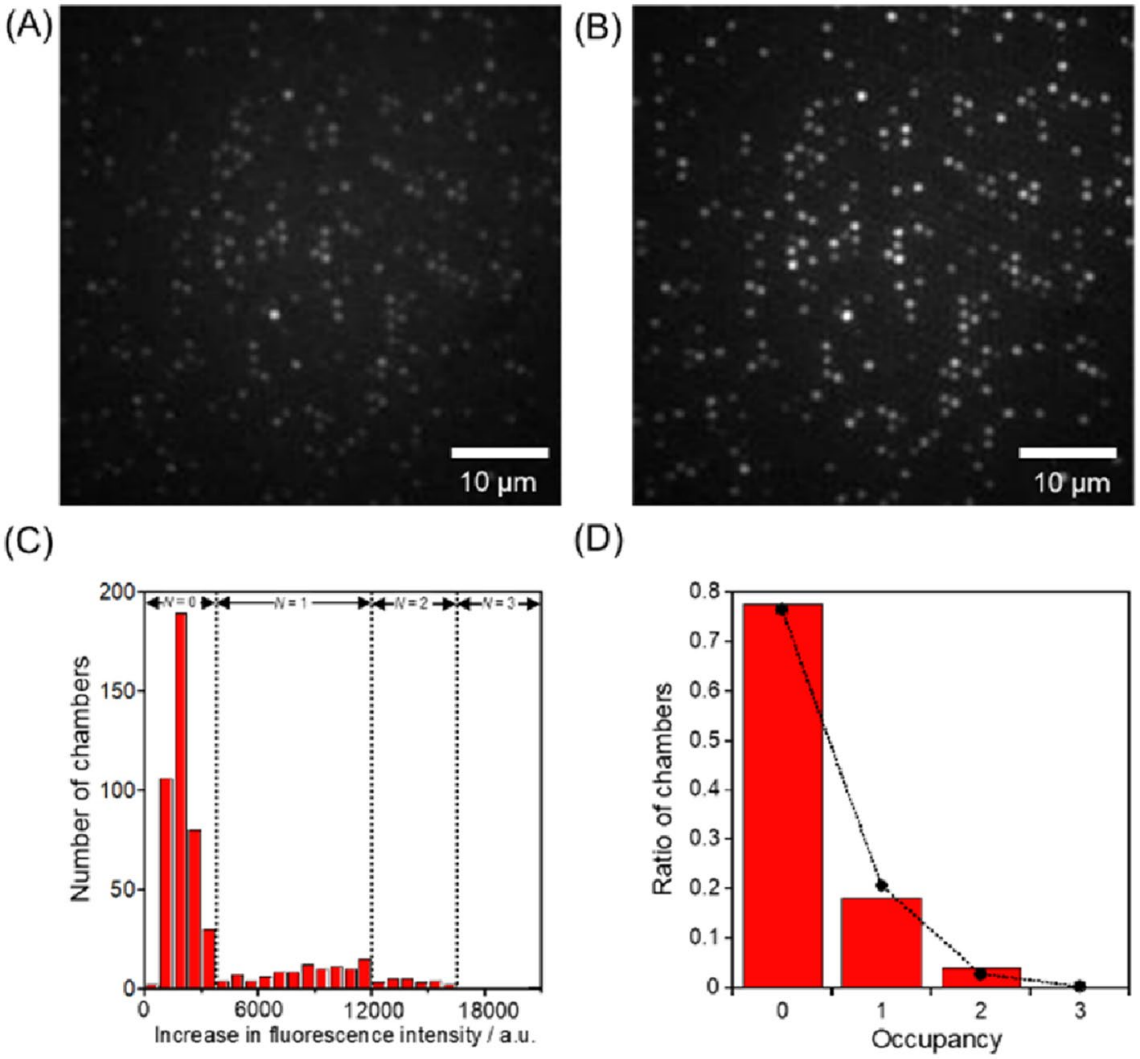
Single-enzyme assay in the 270-aL chambers. The fluorescent images of the microchamber array enclosing *β*-gal after times (**A**) 0 and (**B**) 2 min. (**C**) Histogram of the fluorescent intensity changes for 2 min. The concentrations of *β*-Gal and FDG were 400 ng/mL and 200 μM, respectively. The dotted lines indicate the thresholds of fluorescence intensity increase with different numbers of *β*-gal molecules in the microchambers, assuming a Poisson distributed encapsulation of *β*-gal molecules in the microchamber. (**D**) Occupancy distribution of the microchambers under the condition of 400 ng/mL *β*-gal. The bars show the ratio of the microchambers with an occupancy of *N* enzymes (*N* = 0, 1, 2, 3). The circles indicate the probability of the ratio of the microchambers that were captured *N* enzymes at λ = 0.270, assuming it was a Poisson distribution. The detail explanation is described in the main text. All the fluorescent images were captured under the illumination of a 1.8 μW excitation laser.

Three typical assay results are shown in Figs. 3, 4, and 5 using 624-fL, 61-fL, and 270-aL chambers, respectively. (The other assay results are shown in Figure S3–11.) From the fluorescence images taken 2 min after the start, the increases in fluorescence intensity were measured, and the histograms were obtained. As elucidated in the previous research^17^, the increase in fluorescence intensity can be quantified depending on the number of enclosed *β*-gal. The occupancy probability of the number of enclosed *β*-gal, *X*, can be assumed to follow the Poisson distribution expressed in the following equation;

$${\varvec{X}}={{\varvec{\lambda}}}^{{\varvec{N}}}\times {{\varvec{e}}}^{-{\varvec{\lambda}}}/{\varvec{N}}!$$

where *λ* is the expected number of enzymes trapped in the chamber and *N*! is the factorial of the probability mass function of *X*. When the occupancy distribution approached to the fitted line at *λ* = 1, the observed ratio of chambers at the given concentration of *β*-gal confirmed the trapping of *β*-gal in the microchambers at the single enzyme level. The specific mathematical procedures are the followings: Based on the applied *β*-gal concentration, the occupancy probability of the number of encapsulated *β*-gal in each chamber, *X*, was calculated, assuming that loading process into the chambers followed the Poisson distribution. These probabilities yielded the expected numbers containing 0, 1, 2, and 3 of *β*-gal and were plotted as shown in Figs. 3D, 4D, and 5D. From the plotted ratio of chambers, the number of chambers containing 0, 1, 2, and 3 enzymes were assigned in order of the increase of fluorescence intensity from weakest to strongest. The dotted lines superimposed in Figs. 3C, 4C, and 5C indicate the thresholds of the fluorescence increases containing different numbers of *β*-gal molecules, assuming a Poisson-distributed loading of *β*-gal in the microchambers. Since the fluorescence increase data were classified as different numbers of *β*-gal molecules, the hydrolysis rates per a single *β*-gal molecule were calculated by reference to the fluorescein standard curve.

**Figure 6:**
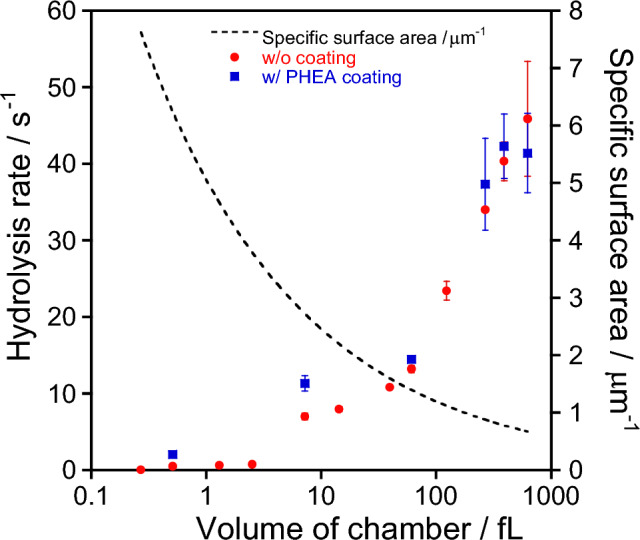
Hydrolysis rate and specific surface area as a function of volume of chamber without or with PHEA coating. The error bars show the standard deviation of the triplicate measurements. Some dots overlapped with the error bars.

The hydrolysis rates,* k*_*cat*_, of a single *β*-gal in 624-fL, 61-fL, and 270-aL chambers were 45.9 ± 7.5 s^–1^, 13.0 ± 0.5 s^–1^, and 0.067 ± 0.0038 s^–1^, respectively. Considering the hydrolysis rates in the bulk experiment, *k*_*cat*_ = 55.1 s^–1^, the largest chamber size of 624 fL gave the similar hydrolysis rates but the other chambers showed that smaller chambers decreased the *β*-gal activity as shown in Fig. 6. The overall tendency seems to be a symmetrical relationship to the specific surface area of the chambers, larger specific surface area decreased the hydrolysis rate. The possible reason could be competitive or non-competitive inhibition by the product, galactose^37,38^. However, all the inhibition experiments were performed with galactose concentrations ranging from a few to several hundred mM in bulk. In contrast, the current single *β*-gal assay system typically uses 200 μM of FDG, and the product galactose should be less than this concentration even when the enzymatic reaction is complete. In addition, the total assay time was less than 5 min after mixing the *β*-gal and FDG. The number of FDG in the microchamber is more than 7.5 × 10^4^ even in the smallest 510 aL chamber. If *β*-gal works as 50 s^-1^, which is a comparable activity to the bulk system, it will take 25 min to consume all the FDG and fill the microchamber with galactose. Therefore, we believe that the majority of the microchamber space is occupied by FDG (not galactose) and that the inhibitory effect of the product, galactose, does not need to be considered during the experimental period of about 5 min.

Another possible reason could be the non-specific adsorption of *β*-gal and FDG on the PDMS chamber surface and the accurate measurements were not achieved. Therefore, to prevent non-specific adsorption on the surface, PHEA was dynamically coated on the PDMS surface and a single *β*-gal activity assay was performed. As shown in Fig. 6, the hydrolysis rates were slightly increased in each chamber, but the overall downward trend was not changed. The second possible reason could be derived from the water structure within the nanometer-scale confinement. As pointed out by Perillo et al*.*, the water structure and dynamics could influence the conformation and the function (net hydrolysis rate) of *β*-gal in the nanoporous structures of a silicate matrix with a mean pore diameter of 33 ± 2 nm^39,40^. Compared to the reaction space in the nanopore, our chamber system has more than 100 times the volume and appears to have less or no influence on *β*-gal conformation by the water structure at the chamber surface. However, if 10–20 nm from the chamber surface could affect the water structure, the diffusional approach of *β*-gal to the vicinity of the chamber surface could change the hydrolysis rate. The residence time near the chamber surface will increase according to the chamber volume reduction, so the downward tendency of *β*-gal hydrolysis rate might be reasonable. In addition, Tsukahara et al. reported faster proton transfer in the 10–100 nm range from the quartz surface, suggesting a long-range influence on water structure than that is originally expected in nanoporous structures^41^. Therefore, we attempted to investigate how the PDMS surface affects the water structure inside the chambers, including proton association and dissociation at the plasma-treated PDMS surface.

**Figure 7:**
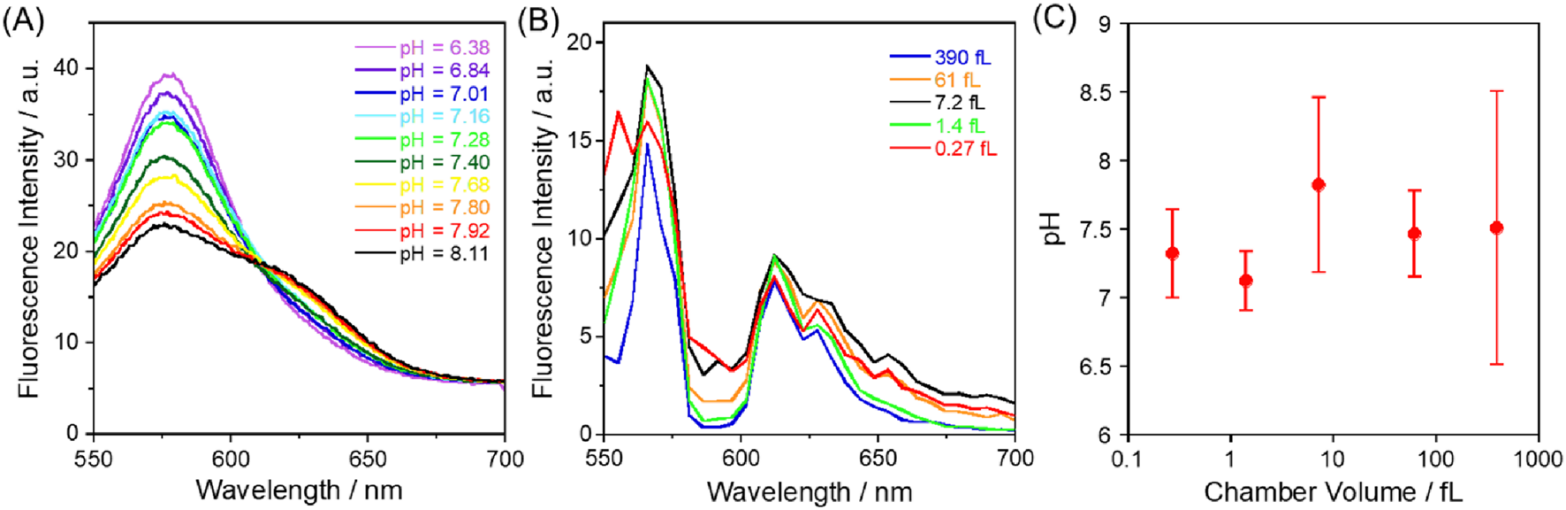
Fluorescence spectra of 5-(and-6)-Carboxy SNARF-1 at 10 µM dissolved in phosphate buffer (pH = 7.4) and excited at 488 nm (**A**) in 1-cm cuvettes and (**B**) microchambers. (**C**) Calculated pH values by the following equation: $\mathrm{pH}={\mathrm{p}K}_{a}-\mathrm{log}\left[\frac{R-{R}_{B}}{{R}_{A}-R}\times \frac{{F}_{B\left(\lambda 2\right)}}{{F}_{A\left(\lambda 2\right)}}\right]$, where *R* is the ratio $\frac{{F}_{\lambda 1}}{{F}_{\lambda 2}}$ of fluorescence intensities measured at two wavelengths. *λ*_*1*_ (568 nm) and *λ*_*2*_ (616 nm), and the subscripts *A* and *B* represent the limiting values at the acidic and basic end points of the titration, respectively. The calibration was performed using a dual-emission ratio with *λ*_*1*_ = 568 nm and *λ*_*2*_ = 616 nm excited at 488 nm. The error bars show the standard deviation of the triplicate measurements.

To evaluate the “free” proton in the chambers, the spectrum changes of the pH indicator, Carboxy SNARF-1, encapsulated in the chambers were observed. As shown in Fig. 7A and B, the spectrum changes from pH = 6.38–8.11 were confirmed in the bulk, and then, the spectrum of PBS adjusted to pH = 7.54 was measured in the different sizes of microchambers. The pH values were calculated using the following equation;

$$\mathbf{p}\mathbf{H}={\mathbf{p}{\varvec{K}}}_{{\varvec{a}}}-\mathbf{log}\left[\frac{{\varvec{R}}-{{\varvec{R}}}_{{\varvec{B}}}}{{{\varvec{R}}}_{{\varvec{A}}}-{\varvec{R}}}\times \frac{{{\varvec{F}}}_{{\varvec{B}}\left({\varvec{\lambda}}2\right)}}{{{\varvec{F}}}_{{\varvec{A}}\left({\varvec{\lambda}}2\right)}}\right]$$

where *R* is the ratio $\frac{{{\varvec{F}}}_{{\varvec{\lambda}}1}}{{{\varvec{F}}}_{{\varvec{\lambda}}2}}$ of fluorescence intensities measured at two wavelengths. *λ*_*1*_ (568 nm) and *λ*_*2*_ (616 nm), and the subscripts *A* and *B* represent the limits at the acidic and basic endpoints of the titration, respectively. Calibration was performed using a dual emission ratio with *λ*_*1*_ = 568 nm and *λ*_*2*_ = 616 nm excited at 488 nm. As shown in Fig. 7C, the result appeared to be very small pH changes in the two smallest chambers, but there was no significant pH difference between the two smaller and three larger chambers. The result could be understood as it is, but the spatial and spectral resolution of the confocal microscope may not be sufficient to resolve the difference in the microchambers. The spatial and spectral resolution of the confocal microscope was about 500 nm and 5 nm, respectively, so the spectrum obtained from each pixel was not enough to recognize the pH distribution within the chambers. Another possible reason is the lack of sensitivity of SNARF-1. In the case of 1 fL chamber, it contains only 60 protons at pH = 7 and 6 protons at pH = 8. In contrast, the pH indicator SNARF-1 contains 600 molecules in 1 fL chamber at 10 μM condition. Therefore, most of the pH indicator did not dissociate or associate depending on the pH, resulting in insufficient sensitivity to detect the pH distribution within the chambers. Further development of the observation system and pH indicator, such as super-resolution microscopy, is expected to solve these problems and reveal the long-range interaction of proton molecules with the chamber surface. This uneven distribution of molecules in nanometer-scale space raised a new question about how biochemical molecules control the reaction kinetics in living cells. Several studies have attempted to unravel biomolecular functions such as gene expression and signal transduction across scales from molecular to cellular scales from the perspectives of biomolecular condensation and intracellular liquid–liquid phase separation^11^. Our micro- and nanochamber system will contribute to the pursuit of biological mechanisms in the confined spaces as an alternative method to liquid droplet systems^42^ and mesoporous systems^43^.

Throughout this entire experiment, the encapsulation probability of *β*-gal molecules in the microchambers was assumed to follow a Poisson distribution. However, the encapsulation process might not occur in a completely independent manner without some specific or biased chemical interaction between the *β*-gal molecules and the PDMS surface. If the encapsulation process followed non-Poisson distribution, such as sub-Poisson or super-Poisson distribution, the obtained hydrolysis rate and the above interpretation would be changed. Therefore, with this opposite approach, an accurate comparison of the statistical interpretation and the experimental results could provide insight into the undetected interaction in this experimental system.

## Conclusions

In this work, a pneumatic valve-based confinement system for micro- and nanochamber arrays was developed to provide technically simple and reproducible single enzyme assay. Using the system, we have experimentally demonstrated that the diffusion-limited confinement space, from micrometer to nanometer cubic, reduced the hydrolysis rates of *β*-gal to about one tenth depending on the reaction space. Since the PHEA-coated chamber surface could not prevent the kinetic tendency, non-specific adsorption of *β*-gal, substrate and product on the large specific surface area may not be the main reason. Another possibility was the silanol group of the PDMS surface dissociation, which could affect the proton localization in the solution and change the dissociation state of *β*-gal. The optical resolution was not sufficient to prove the hypothesis, but the micro- and nanochamber array system showed quantitative results even in such a small space from micro- to nanometer cubic scale. The proposed experimental system will help to mimic and analyze recent challenging topics such as liquid–liquid phase separation and biomolecular condensates in a cell^44^.

### Supplementary Information

Supplementary Information 1.

Supplementary Information 2.

Supplementary Video 1.

## Data Availability

The datasets used and/or analyzed during the current study available from the corresponding author on reasonable request.
