# Supplementary material for: Micro- and nanochamber array system for single enzyme assays
Source: Sci Rep. 2023 Aug 16;13:13322. doi: 10.1038/s41598-023-40544-4 (PMC10432523; doi:10.1038/s41598-023-40544-4)
Supplement: Supplementary file 2 — Supplementary Information 2. [file 41598_2023_40544_MOESM2_ESM.docx]

Electronic Supplementary Information

Micro- and nanochamber array system for single enzyme assays

Kazuki Iijima^1^, Noritada Kaji*^2,3^, Manabu Tokeshi ^3,4^ and Yoshinobu Baba ^1,3,5,6^

1. Department of Biomolecular Engineering, Graduate School of Engineering, Nagoya University, Furo-cho, Chikusa-ku, Nagoya 464-8603, Japan.
2. Department of Applied Chemistry, Graduate School of Engineering, Kyushu University, 744 Motooka, Nishi-ku, Fukuoka 819-0395, Japan.
3. Institute of Nano-Life-Systems, Institutes of Innovation for Future Society, Nagoya University, Furo-cho, Chikusa-ku, Nagoya 464-8603, Japan.
4. Division of Applied Chemistry, Faculty of Engineering, Hokkaido University, Kita-13, Nishi-8, Kita-Ku, Sapporo 060-8628, Japan
5. Institute for Quantum Life Science, Quantum Life and Medical Science Directorate, National Institutes for Quantum Science and Technology, Chiba, 263-8555, Japan
6. School of Pharmacy, College of Pharmacy, Kaohsiung Medical University, 100, Shih-Chuan 1st Rd., Kaohsiung, 807, Taiwan, R.O.C.
7. SEM images of the micro and nanochambers array.
8. Enzyme reaction kinetics in the bulk.
9. Single-enzyme assay in the 390-fL chambers.
10. Single-enzyme assay in the 264-fL chambers.
11. Single-enzyme assay in the 123-fL chambers.
12. Single-enzyme assay in the 39-fL chambers.
13. Single-enzyme assay in the 14-fL chambers.
14. Single-enzyme assay in the 7.2-fL chambers.
15. Single-enzyme assay in the 2.5-fL chambers.
16. Single-enzyme assay in the 1.3-fL chambers.
17. Single-enzyme assay in the 510-aL chambers.

Movie S1: Real-time observation of the trapping process of Fluorescein solution in the 264-fL chambers. The movie shows the time course of sealing process of the microchambers by the pneumatic valve, where the microchambers layer gradually approached to the PDMS-coated glass surface at the focus of the microscope objective lens. According to the increase of the liquid pressure in the control channel, the fluorescence intensity of fluorescein solution transiently increased, and then, microchambers successfully enclosed the solution and the other regions than the microchambers extruded the excess liquid in the microchambers.


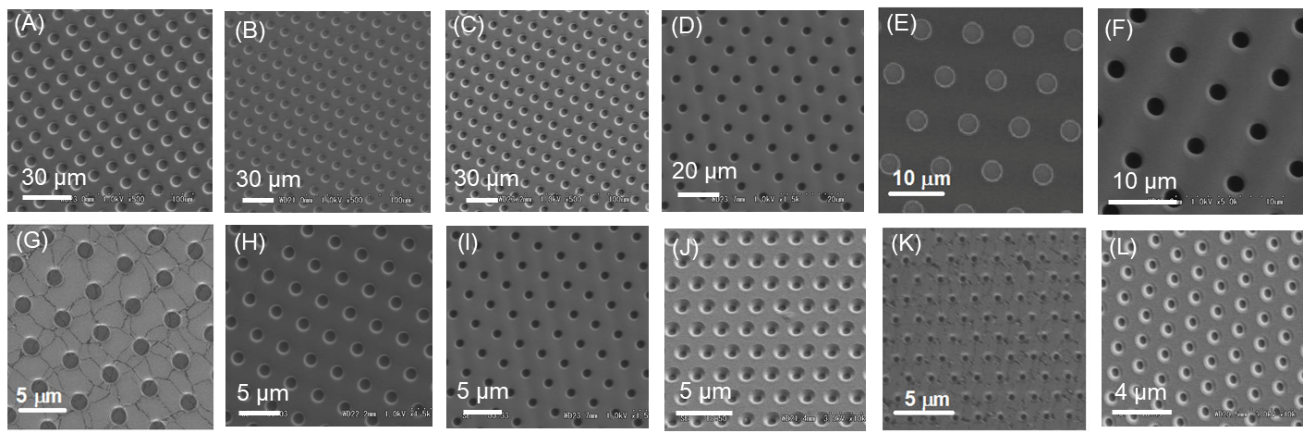


Figure S1. SEM images of the micro and nanochambers array. (A) 624 fL (*ϕ*:10 µm × *H*:10 µm), (B) 390 fL (*ϕ*:10 µm × *H*:6.5 µm), (C) 264 fL (*ϕ*:9.1 µm × *H*:6.3 µm), (D) 123 fL (*ϕ*:6.7 µm × *H*:6.3 µm), (E) 61 fL (*ϕ*:4.0 µm × *H*:5.1 µm), (F) 39 fL (*ϕ*:4.0 µm × *H*:4.3 µm), (G) 7.2 fL (*ϕ*:2.4 µm × *H*:1.6 µm), (H) 4.6 fL (*ϕ*:2.0 µm × *H*:1.7 µm), (I) 2.5 fL (*ϕ*:1.4 µm × *H*:1.6 µm), (J) 1.3 fL (*ϕ*:1.3 µm × *H*:1.1 µm), (K) 510 aL (*ϕ*:850 nm × *H*:900 nm), (L) 270 aL (*ϕ*:800 nm × *H*:600 nm).

Figure S2. Enzyme reaction kinetics of *β*-gal in the bulk at 30˚C. Time course of the fluorescence intensities of the produced fluorescein at different FDG concentrations from 50 to 200 μM.


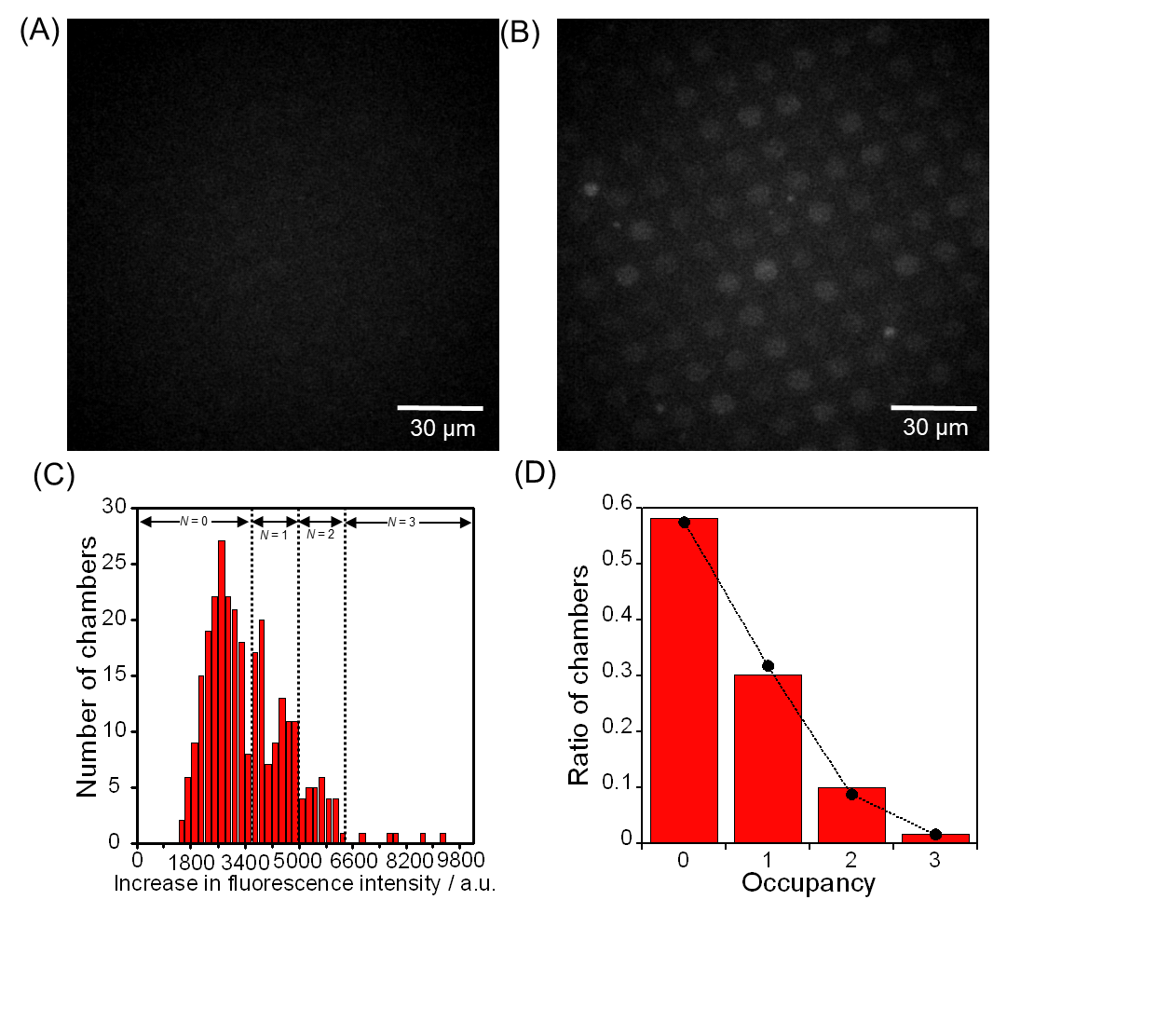
Figure S3. Single-enzyme assay in the 390-fL chambers. The fluorescent images of the microchamber array enclosing *β*-gal after times (A) 0 and (B) 4 min. (C) Histogram of the fluorescent intensity changes for 4 min. The concentrations of *β*-Gal and FDG were 1.0 ng/mL and 200 μM, respectively. (D) Occupancy distribution of the microchambers under the condition of 1.0 ng/mL *β*-gal. The bars show the ratio of the microchambers with an occupancy of N enzymes (N = 0, 1, 2, 3). The circles indicate the probability of the ratio of the microchambers that were captured N enzymes at λ = 0.553, assuming it was a Poisson distribution. The detail explanation is described in the main text. All the fluorescent images were captured under the illumination of a 60.5 μW excitation laser.


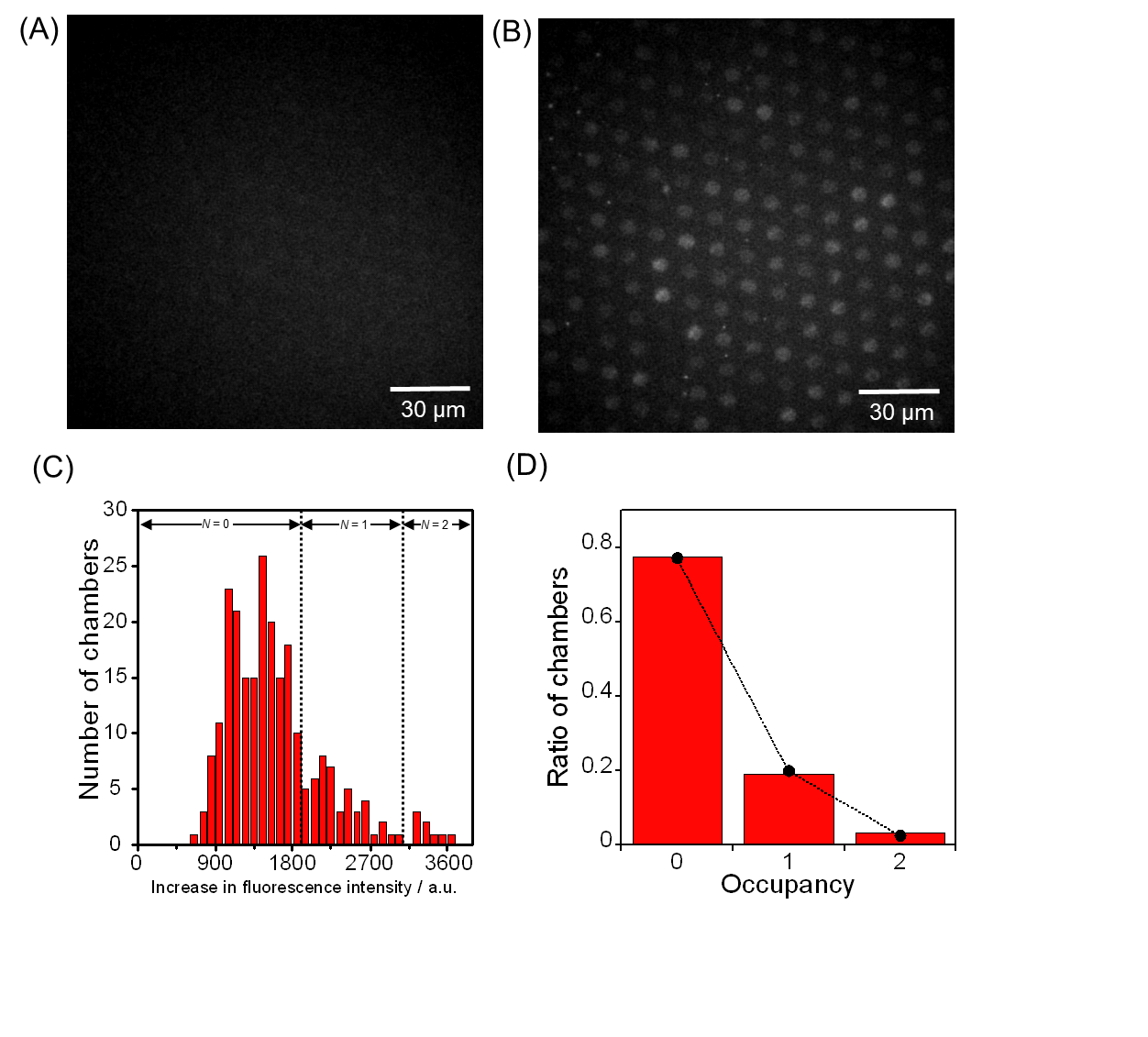
Figure S4. Single-enzyme assay in the 264-fL chambers. The fluorescent images of the microchamber array enclosing *β*-gal after times (A) 0 and (B) 4 min. (C) Histogram of the fluorescent intensity changes for 4 min. The concentrations of *β*-Gal and FDG were 2.0 ng/mL and 200 μM, respectively. (D) Occupancy distribution of the microchambers under the condition of 2.0 ng/mL *β*-gal. The bars show the ratio of the microchambers with an occupancy of N enzymes (N = 0, 1, 2). The circles indicate the probability of the ratio of the microchambers that were captured N enzymes at λ = 0.258, assuming it was a Poisson distribution. The detail explanation is described in the main text. All the fluorescent images were captured under the illumination of a 60.5 μW excitation laser.


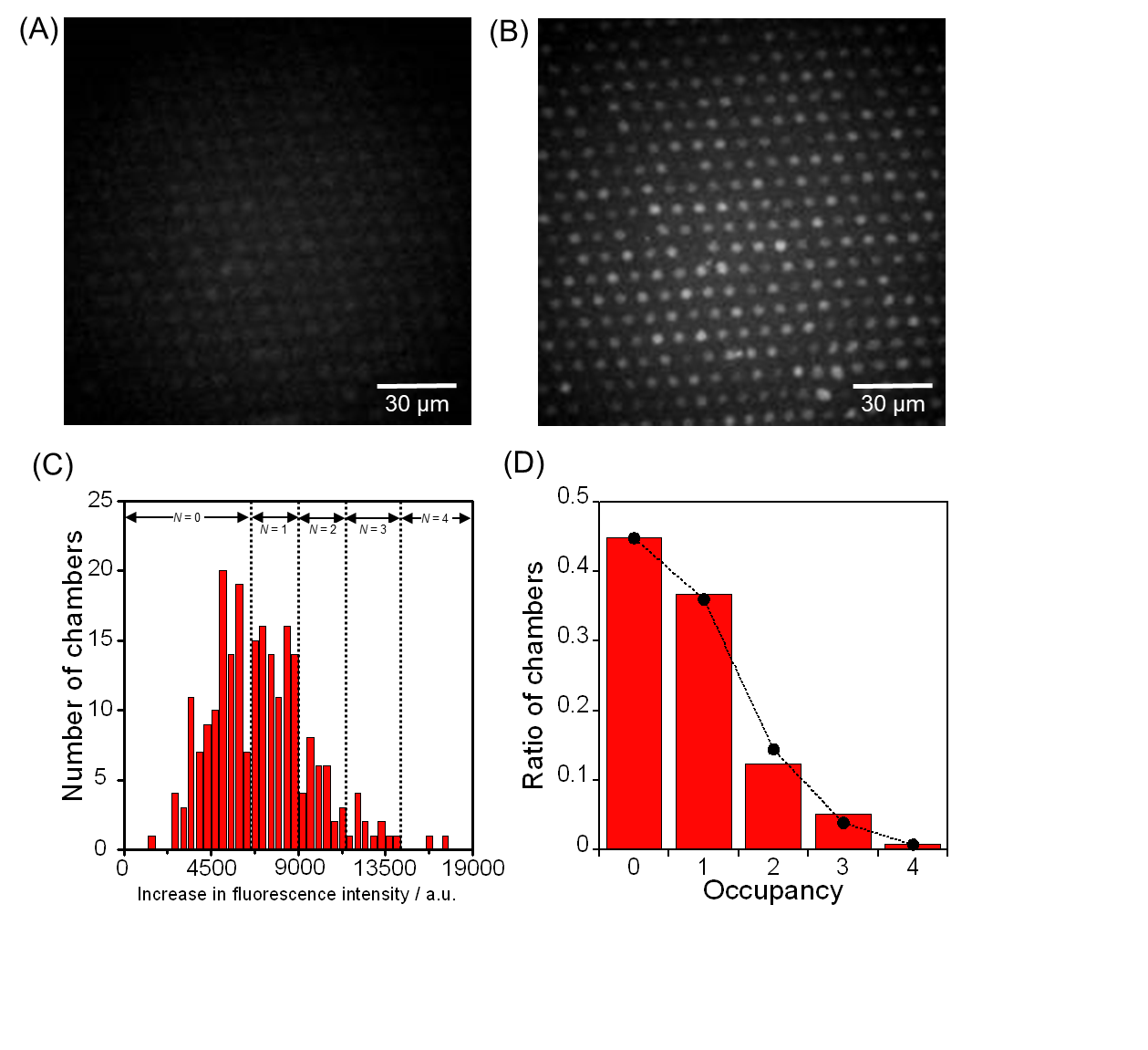
Figure S5. Single-enzyme assay in the 123-fL chambers. The fluorescent images of the microchamber array enclosing *β*-gal after times (A) 0 and (B) 4 min. (C) Histogram of the fluorescent intensity changes for 4 min. The concentrations of *β*-Gal and FDG were 1.0 ng/mL and 200 μM, respectively. (D) Occupancy distribution of the microchambers under the condition of 1.0 ng/mL *β*-gal. The bars show the ratio of the microchambers with an occupancy of N enzymes (N = 0, 1, 2, 3, 4). The circles indicate the probability of the ratio of the microchambers that were captured N enzymes at λ = 0.803, assuming it was a Poisson distribution. The detail explanation is described in the main text. All the fluorescent images were captured under the illumination of a 60.5 μW excitation laser.


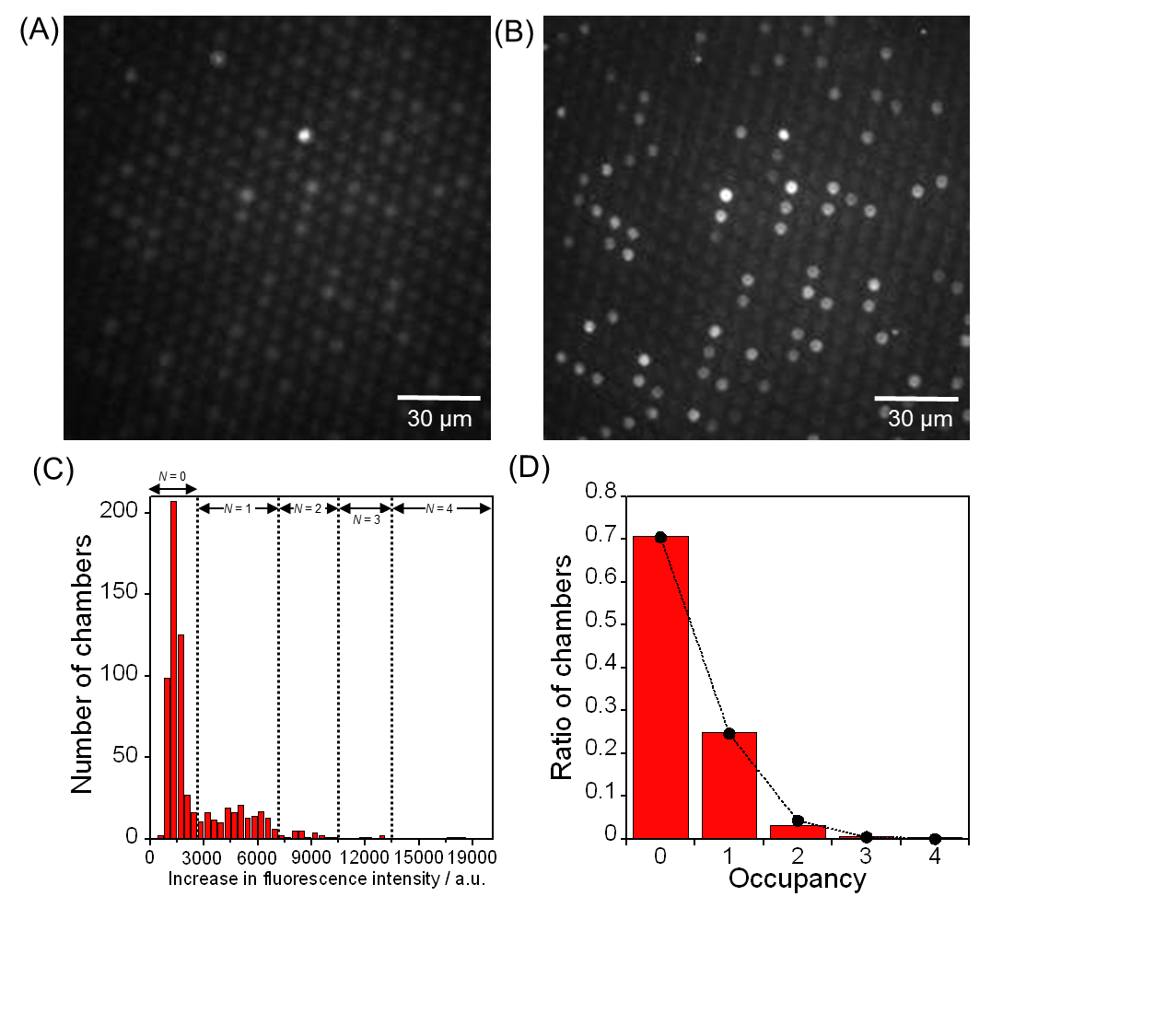
Figure S6. Single-enzyme assay in the 39-fL chambers. The fluorescent images of the microchamber array enclosing *β*-gal after times (A) 0 and (B) 2 min. (C) Histogram of the fluorescent intensity changes for 2 min. The concentrations of *β*-Gal and FDG were 3.3 ng/mL and 200 μM, respectively. (D) Occupancy distribution of the microchambers under the condition of 3.3 ng/mL *β*-gal. The bars show the ratio of the microchambers with an occupancy of N enzymes (N = 0, 1, 2, 3, 4). The circles indicate the probability of the ratio of the microchambers that were captured N enzymes at λ = 0.351, assuming it was a Poisson distribution. The detail explanation is described in the main text. All the fluorescent images were captured under the illumination of a 20.4 μW excitation laser.


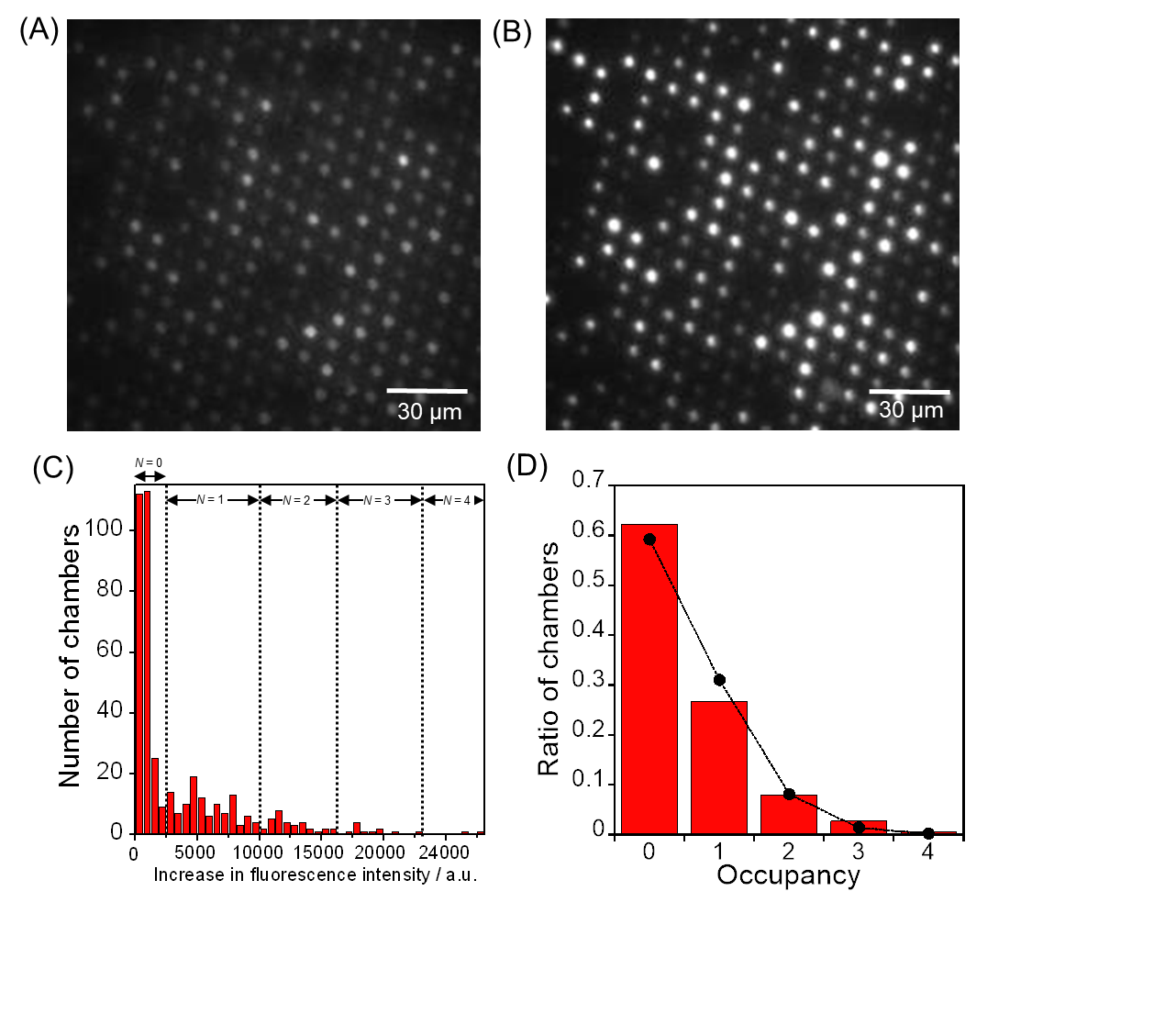
Figure S7. Single-enzyme assay in the 14-fL chambers. The fluorescent images of the microchamber array enclosing *β*-gal after times (A) 0 and (B) 2 min. (C) Histogram of the fluorescent intensity changes for 2 min. The concentrations of *β*-Gal and FDG were 20 ng/mL and 200 μM, respectively. (D) Occupancy distribution of the microchambers under the condition of 20 ng/mL *β*-gal. The bars show the ratio of the microchambers with an occupancy of N enzymes (N = 0, 1, 2, 3, 4). The circles indicate the probability of the ratio of the microchambers that were captured N enzymes at λ = 0.524, assuming it was a Poisson distribution. The detail explanation is described in the main text. All the fluorescent images were captured under the illumination of a 17.8 μW excitation laser.


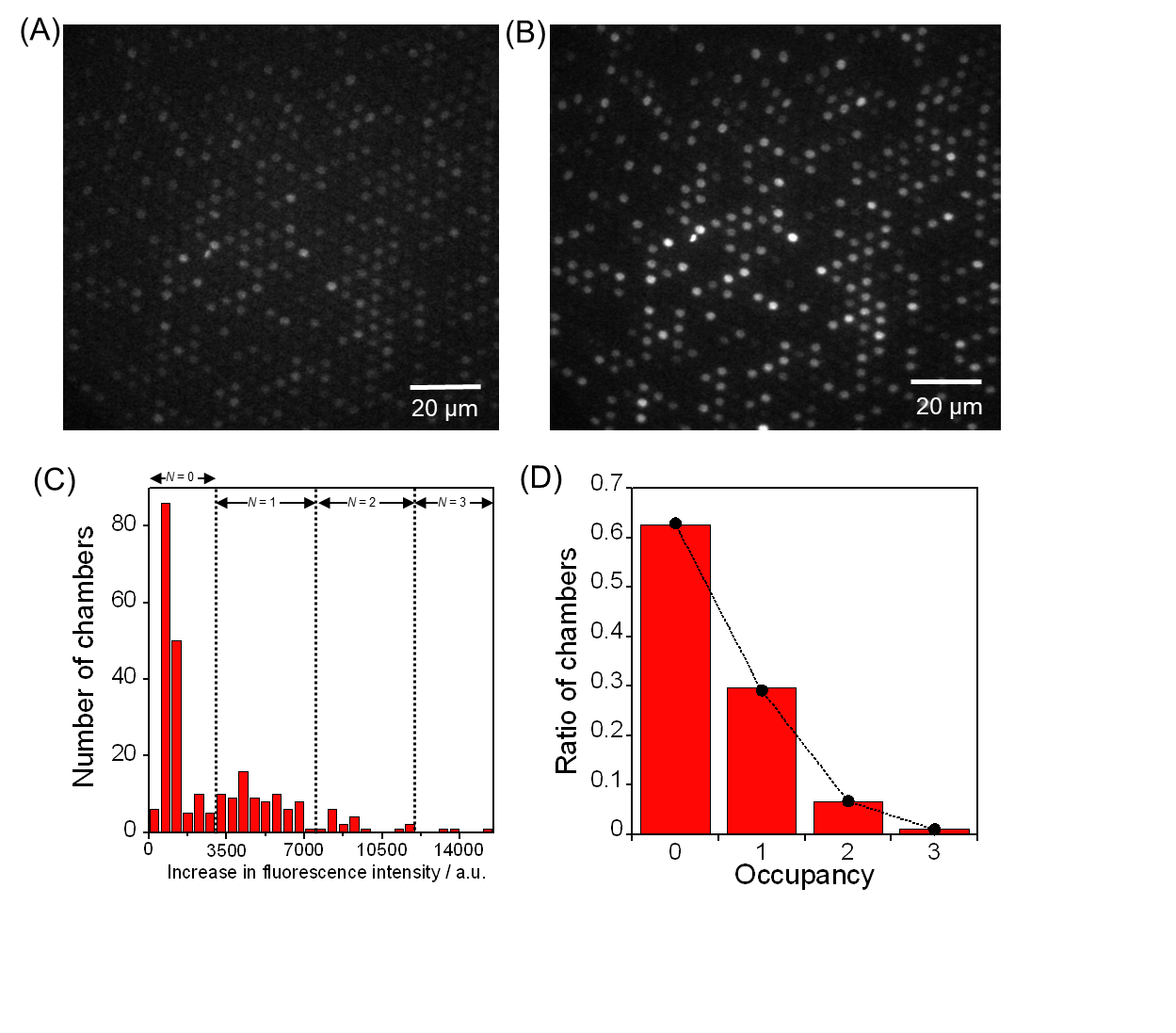
Figure S8. Single-enzyme assay in the 7.2-fL chambers. The fluorescent images of the microchamber array enclosing *β*-gal after times (A) 0 and (B) 2 min. (C) Histogram of the fluorescent intensity changes for 2 min. The concentrations of *β*-Gal and FDG were 10 ng/mL and 200 μM, respectively. (D) Occupancy distribution of the microchambers under the condition of 10 ng/mL *β*-gal. The bars show the ratio of the microchambers with an occupancy of N enzymes (N = 0, 1, 2, 3). The circles indicate the probability of the ratio of the microchambers that were captured N enzymes at λ = 0.463, assuming it was a Poisson distribution. The detail explanation is described in the main text. All the fluorescent images were captured under the illumination of a 7.8 μW excitation laser.


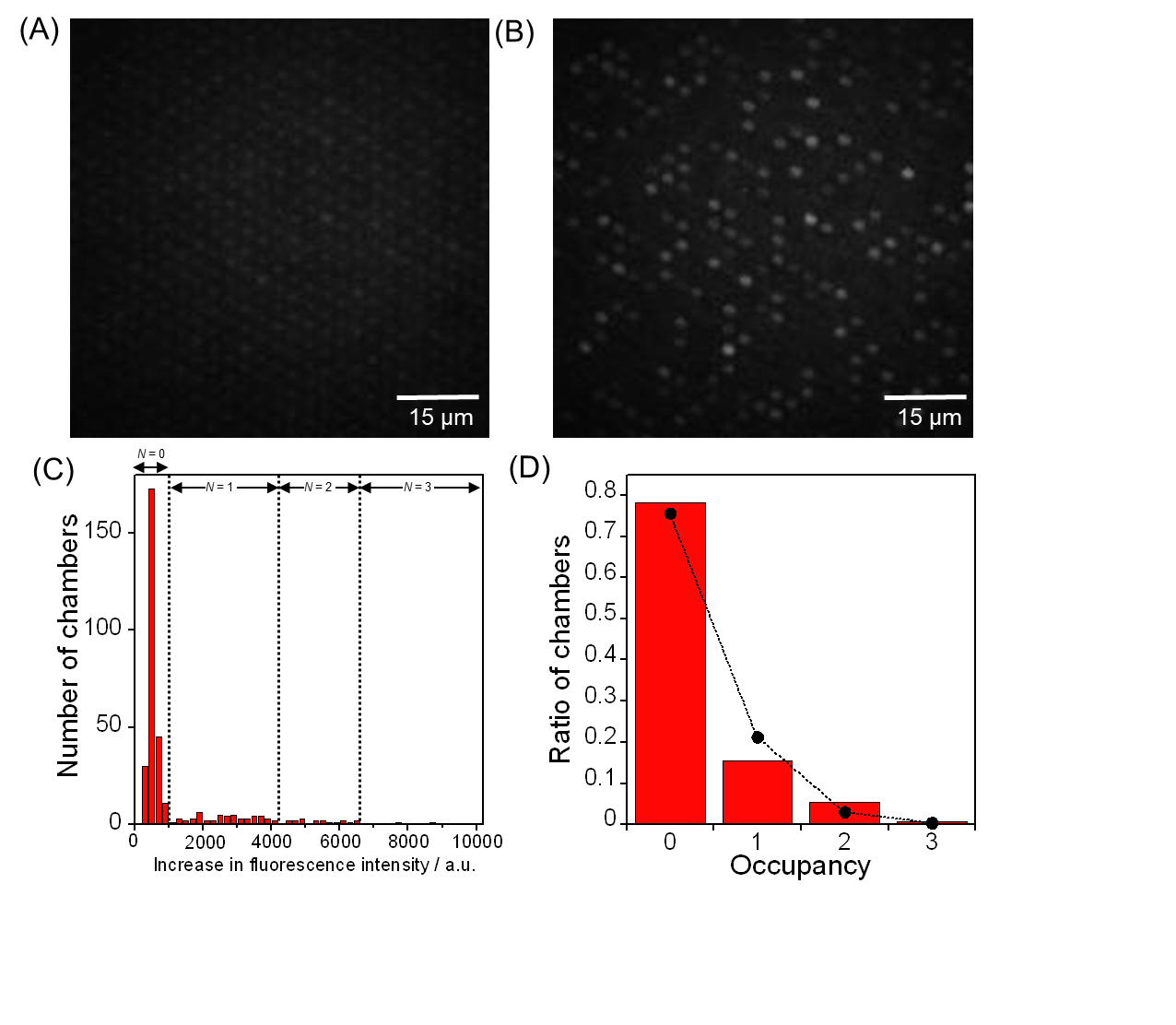
Figure S9. Single-enzyme assay in the 2.5-fL chambers. The fluorescent images of the microchamber array enclosing *β*-gal after times (A) 0 and (B) 2 min. (C) Histogram of the fluorescent intensity changes for 2 min. The concentrations of *β*-Gal and FDG were 48 ng/mL and 200 μM, respectively. (D) Occupancy distribution of the microchambers under the condition of 48 ng/mL *β*-gal. The bars show the ratio of the microchambers with an occupancy of N enzymes (N = 0, 1, 2, 3). The circles indicate the probability of the ratio of the microchambers that were captured N enzymes at λ = 0.281, assuming it was a Poisson distribution. The detail explanation is described in the main text. All the fluorescent images were captured under the illumination of a 5.1 μW excitation laser.


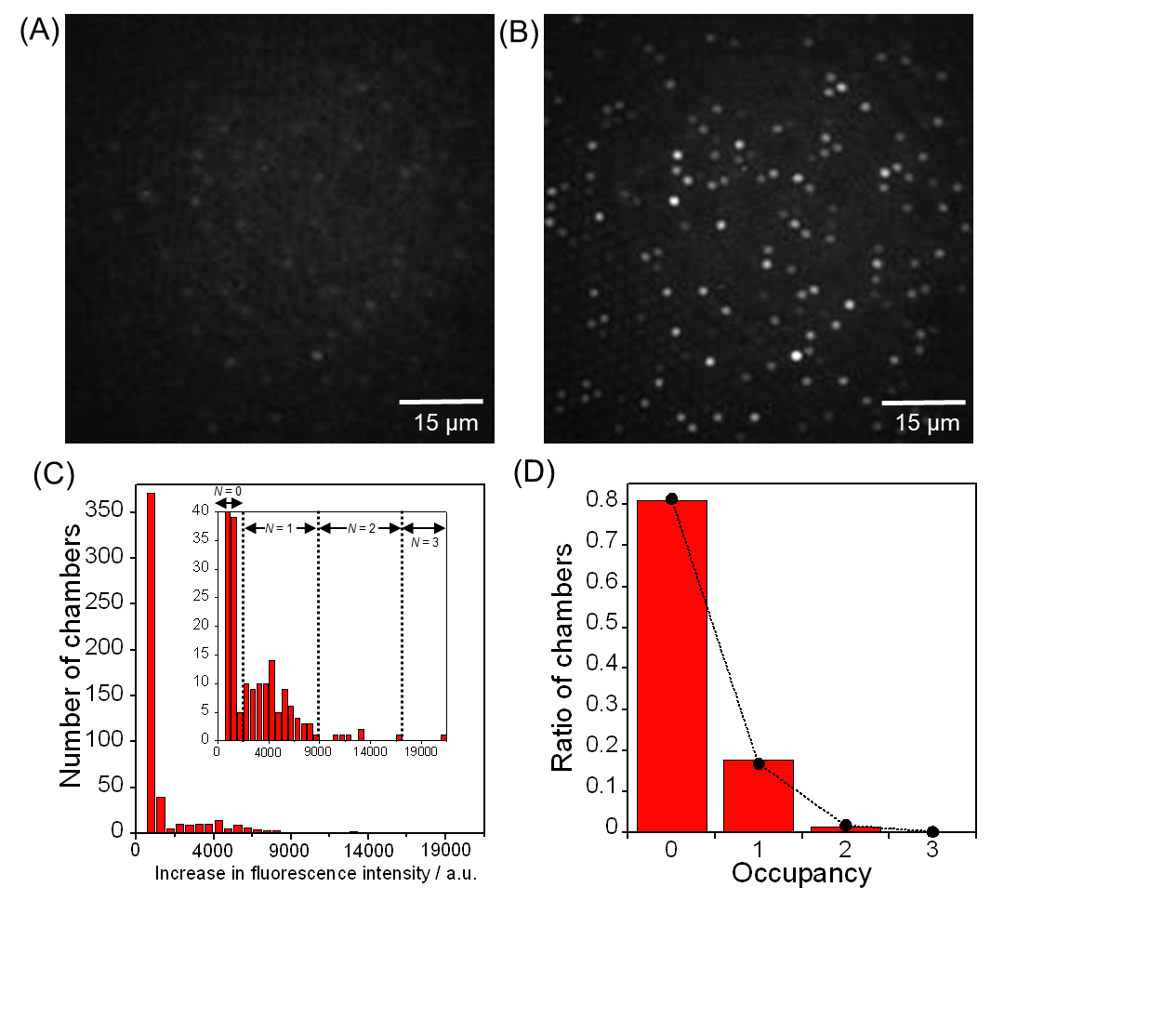
Figure S10. Single-enzyme assay in the 1.3-fL chambers. The fluorescent images of the microchamber array enclosing *β*-gal after times (A) 0 and (B) 2 min. (C) Histogram of the fluorescent intensity changes for 2 min. The concentrations of *β*-Gal and FDG were 48 ng/mL and 200 μM, respectively. The inserted figure is the expanded image along y-axis of (C). (D) Occupancy distribution of the microchambers under the condition of 48 ng/mL *β*-gal. The bars show the ratio of the microchambers with an occupancy of N enzymes (N = 0, 1, 2, 3). The circles indicate the probability of the ratio of the microchambers that were captured N enzymes at λ = 0.206, assuming it was a Poisson distribution. The detail explanation is described in the main text. All the fluorescent images were captured under the illumination of a 4.7 μW excitation laser.


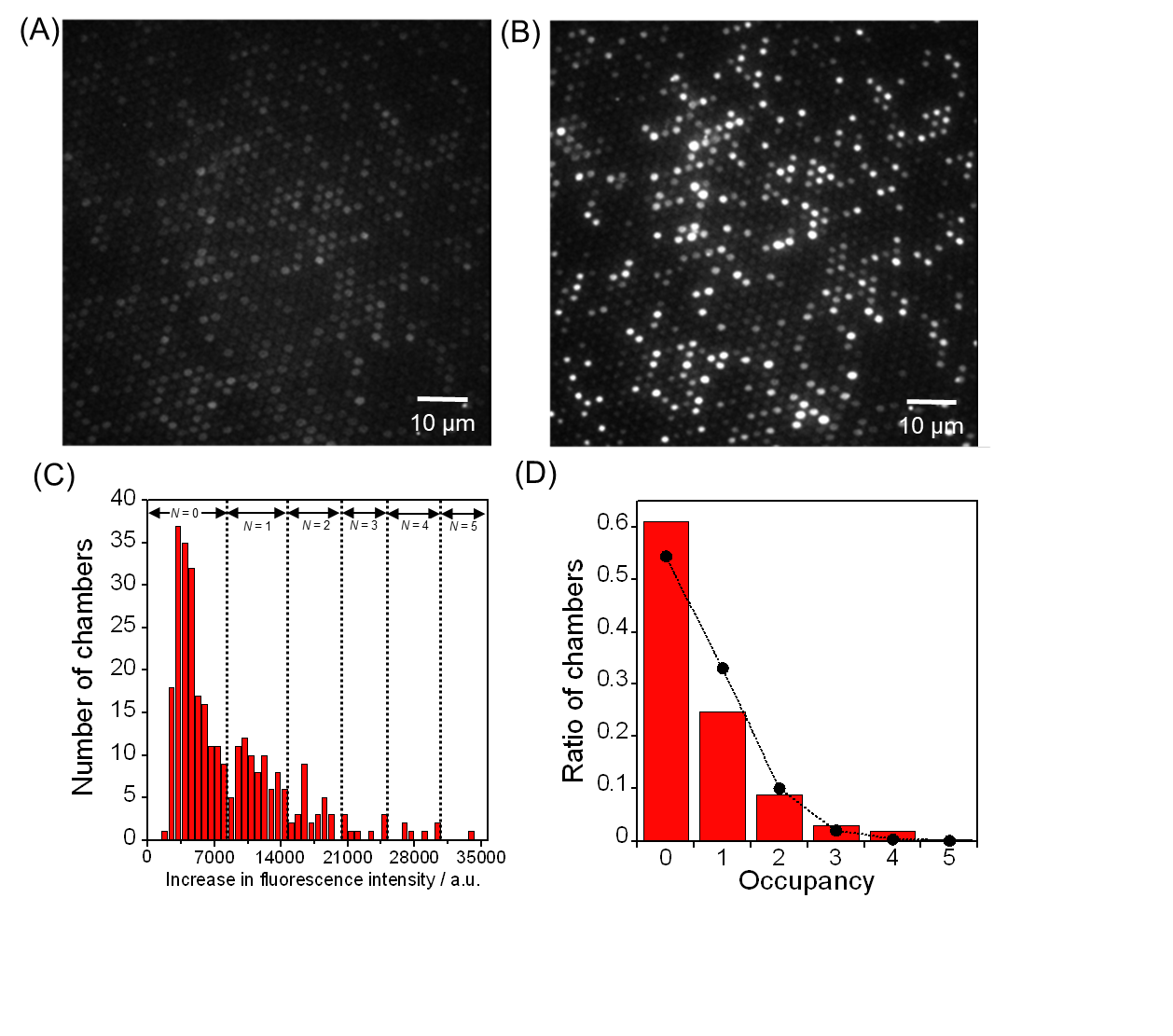
Figure S11. Single-enzyme assay in the 510-aL chambers. The fluorescent images of the microchamber array enclosing *β*-gal after times (A) 0 and (B) 2 min. (C) Histogram of the fluorescent intensity changes for 2 min. The concentrations of *β*-Gal and FDG were 400 ng/mL and 200 μM, respectively. (D) Occupancy distribution of the microchambers under the condition of 400 ng/mL *β*-gal. The bars show the ratio of the microchambers with an occupancy of N enzymes (N = 0, 1, 2, 3, 4, 5). The circles indicate the probability of the ratio of the microchambers that were captured N enzymes at λ = 0.608, assuming it was a Poisson distribution. The detail explanation is described in the main text. All the fluorescent images were captured under the illumination of a 1.7 μW excitation laser.
